# Discrete diffusion models to study the effects of *Mg*^2+^ concentration on the PhoPQ signal transduction system

**DOI:** 10.1186/1471-2164-11-S3-S3

**Published:** 2010-12-01

**Authors:** Preetam Ghosh, Samik Ghosh, Kalyan Basu, Sajal K Das, Chaoyang Zhang

**Affiliations:** 1 Computational Biology and Bioinformatics Lab, School of Computing, The University of Southern Mississippi, USA.; 2 Department of Computer Science and Engineering, The University of Texas at Arlington, USA.

## Abstract

**Background:**

The challenge today is to develop a modeling and simulation paradigm that integrates structural, molecular and genetic data for a quantitative understanding of physiology and behavior of biological processes at multiple scales. This modeling method requires techniques that maintain a reasonable accuracy of the biological process and also reduces the computational overhead. This objective motivates the use of new methods that can transform the problem from energy and affinity based modeling to information theory based modeling. To achieve this, we transform all dynamics within the cell into a random event time, which is specified through an information domain measure like probability distribution. This allows us to use the “in silico” stochastic event based modeling approach to find the molecular dynamics of the system.

**Results:**

In this paper, we present the discrete event simulation concept using the example of the signal transduction cascade triggered by extra-cellular *Mg*^2+^ concentration in the two component PhoPQ regulatory system of Salmonella Typhimurium. We also present a model to compute the information domain measure of the molecular transport process by estimating the statistical parameters of inter-arrival time between molecules/ions coming to a cell receptor as external signal. This model transforms the diffusion process into the information theory measure of stochastic event completion time to get the distribution of the *Mg*^2+^ departure events. Using these molecular transport models, we next study the in-silico effects of this external trigger on the PhoPQ system.

**Conclusions:**

Our results illustrate the accuracy of the proposed diffusion models in explaining the molecular/ionic transport processes inside the cell. Also, the proposed simulation framework can incorporate the stochasticity in cellular environments to a certain degree of accuracy. We expect that this scalable simulation platform will be able to model more complex biological systems with reasonable accuracy to understand their temporal dynamics.

## Introduction

Advancement in high-throughput biological experiments has generated huge amounts of empirical data on the molecular foundations of biological structures and functions that require computer models for analysis. The next challenge is to understand the complex interactions of biological processes and functions creating the intelligence of life. The complexity increases manifold as we move into higher scales: interaction of large ensemble of cells in a tissue or interaction of tissues in an organ. Thus, we need to develop a comprehensive model integrating molecular and genetic data for quantitative studies of physiology and behavior of biological processes at multiple scales [1].

Existing models used in the understanding of biological processes can be divided into three main classes. Quantum Mechanics based models (femtosecond-picosecond; ) are used to understand the structure of macro molecules. The functional understanding of biological molecules (like binding properties, configuration states after binding and other individual properties of the molecular reactions) are well studied by the Molecular dynamics based model (picosecond-nanosecond; 1nm-10nm). The next challenge is to understand the biological intelligence created by the usage of the macro molecules in the cell. These are accomplished by the Mesoscale Dynamics (nanosecond-seconds; 10nm-1mm) models, and Cellular-level/Organ-level simulation schemes. Such simulation schemes are again broadly classified into two categories: (a) Continuous system models [2-6], employing differential equations to simulate cellular dynamics used in tools like Dizzy [6] and JARNAC [3]; (b) Stochastic discrete time models, like StochSim [7] and M-cell [8], that have been developed for capturing the stochastic nature of molecular interactions within the existing framework of rate equations in continuous time domain. Most of these models focus on intracellular biochemical reactions and require accurate estimation of a very large number of system parameters for providing systemic understanding of underlying processes. More integrative tools at the whole cell level have also been developed, which try to model cellular mechanisms and present visual representation of their functionality [9,10].

Recently, it was shown that gene expression type interactions create a stochastic resonance [11] within the system and hence deterministic models are inappropriate for this process. The Gilliespie simulation [12] incorporates the dynamics of the chemical master equation by approximately handling the stochasticity of the mass-kinetic equations. As the temporal variability of reaction time is appreciable within a biological process, this method suffers from simulation stiffness. Moreover, this model represents the biological pathways through a set of reaction equations without showing their relation to the biological functions creating the pathway. Any change in the pathway description may change the complete set of equations. Also their approach has computational overheads and require estimates of all the rate constants. With the existing systems in perspective, we present a discrete-event driven paradigm - modeling a composite system by combining the state variables in the time-space domain as events and determining the immediate dynamics between the events by using statistical analysis or simulation methods. To reduce computational overhead we transform the different diffusion and molecular interactions within a cell from the thermodynamic energy based fields to the information theory field by suitable abstraction of the energy field profiles into selected statistical distributions. Our goals are to [13][14]: (1) Use the results of the Quantum Mechanics based models (molecular structure data) and Molecular dynamics models (molecular binding data) to create the micro-level biological event models. (2) Transform the energy driven biological effects to information theory parameters in the probabilistic domain considering the biological functions. (3) Develop event models to estimate the statistics of the biological event. (4) Develop a discrete-event based “in silico” simulation for complex systems.

The rest of the paper is organized as follows. We briefly present an overview on the different modeling and simulation paradigms in Section Modeling and Simulation landscape. Next, we introduce the discrete event based simulation framework in Section Discrete Event Simulation Technique. In Section PhoPQ Biological System Model, we present the concept of event abstraction of biological pathways using the PhoPQ biological system in Salmonella. We introduce the analytical models for the molecular transport mechanisms in Section Analytical Models for Molecular Transport. In Section Numerical Results for the Molecular Transport Models, we present the performance results and validation of the molecular transport models. Section Simulation Results of the PhoPQ system presents some in silico results from the discrete event simulation of the PhoPQ system, to validate the performance of the simulation engine and also provides some examples of in silico hypothesis tests. Finally, we conclude in Section Conclusion and provide some directions for future work. A short version of this paper appeared in [15].

### Modeling and simulation landscape

The inherent complexity involved in the molecular processes governing life has motivated the development of computational modeling and simulation techniques to decipher their ensemble dynamics. In this section, we provide an overview of the wide spectrum of in silico modeling and simulation methodologies available for system-wide study of biological processes.

Mathematical models have being extensively used for intracellular molecular networks like kinase cascades and metabolic pathways, gene regulatory networks and protein interaction networks. A large section of the work in computational models of biological systems is based on classical chemical kinetic (CCK) formalism based on a set of ordinary differential equations (ODE), also known as reaction rate equations or mass action kinetics [16]. Representing a homogeneous biological system as a set of biochemical reactions, the temporal dynamics of the molecular species is studied in the continuous-deterministic domain. A large number of computational tools, which provide a software platform for building, storing and parameterizing a set of biochemical reactions and solving those using numerical techniques, are available, like Gepasi [2], Jarnac [3], CyberCell [8], Promot /DIVA [17], Stode [18]. These rate-based models have been successfully applied to study gene expression and other molecular reaction systems.

While continuous-deterministic reaction models are capable of capturing behavioral dynamics for spatially homogeneous systems with large number of molecular species, the inherent stochasticity observed in many biological processes (gene expression and protein synthesis) have proven the limitation of CCK in accurately representing biological processes. In a recent article [16], Arkin et.al have shown the limitations of CCK in several common biological scenarios, where stochastic reaction dynamics present a more accurate picture of the systems behavior. Stochastic models, which present an accurate approximation for the chemical master equation (CME), have been developed, largely based on Gillespie’s algorithm [12,20,12]. In this method, the next reaction event and the time associated with it are computed based on a probability distribution (Monte Carlo Step). Stochastic tools, like StochSim [7], have been developed based on Gillespie’s technique and its computationally efficient variants like Gibson-Bruck [21] and tau-leaping [22-24]. A large number of tools, which provide an integrative environment to build and study biochemical reaction systems in an exchangeable format (like Systems Biology Markup Language (SBML)) using deterministic as well as stochastic techniques are available, like E-Cell [25], Virtual Cell [26], Dizzy [6], CyberCell [27], and M-Cell [28]. These techniques are based on treating a biological process as a system of equations, represented by their rate constants and other parameters (like volume, cell density etc.) and simulating their interactions through numerical techniques or Monte Carlo based stochastic simulations.

Another technique in building abstract computational models for biosimulation has been developed based on Petri nets [29-31] and stochastic process algebra [32]. These methods present a mathematical formalism for representing biochemical pathways within and between cells. In [30], the authors present a stochastic Petri net (SPN) model for studying simple chemical reactions (SPN model of ColE1 plasmid replication) and show how existing softwares can be used to perform structural analysis based on numerical techniques. Discrete event system specifications based on Devs &amp; StateCharts [33] and Stochastic π calculus [34] have been successfully demonstrated to provide a computational platform for temporal simulation of complex biological systems. Hillston et. al have developed a mathematical technique, Performance Evaluation Process Algebra (PEPA) [32], wherein functionality is captured at the level of pathways rather than molecules and the system is represented as a continuous time Markov chain.

Other simulation methodologies, based on object oriented and agent based (ABM) paradigms have also been studied for in silico modeling of complex bio-processes by Uhramacher et.al [35-37]. In [38], the authors have developed AgentCell, an ABM based digital assay for the study of bacterial chemotaxis. Simulation platforms, based on discrete events, where the events are modeled on rate constants and measured experimental data, have been demonstrated in [9,39].

The overarching theme, guiding the development of in silico modeling and simulation tools, is developing models based on continuous-deterministic ODEs or using stochastic simulation algorithms (SSA) for approximating the chemical master equation, which capture the temporal evolution of the biological process dynamics. Most of these techniques focus on molecular pathways, which are represented in graphical and mathematical formalisms, treat spatial dynamics in terms of well-defined cellular compartments, and abstract the complexity in terms of estimated parameters and rate constants. In the next section, we briefly outline our modeling and simulation technique, based on a discrete event system specification, where the molecular events (representing reactions, molecular/ionic transport etc) are mechanistically modeled depending on their biophysical characteristics to compute the probability distribution of their execution times. A discrete event simulation system then links the biological processes to simulate the behavior emerging from the interaction of the events in time.

### Discrete event simulation technique

In a discrete-event based approach, the dynamics of the system are captured by discrete time-space state variables, where an event is a combined process of large number of state transitions between a set of state variables accomplished within event execution time. The underlying assumption is that it is possible to segregate the complete state-space into such disjoint sets of independent events which can execute simultaneously without any interaction.

**Figure 1:**
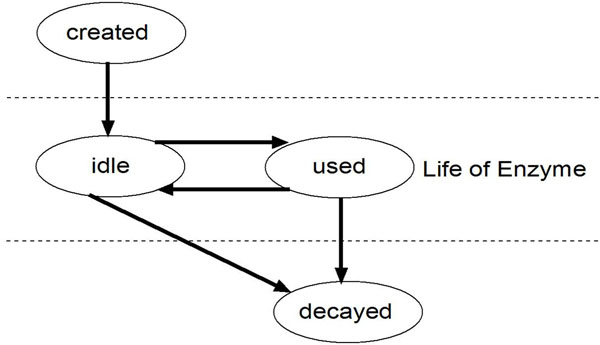
**State transition diagram of an enzyme during its life cycle** A biological process is visualized as a system of resources periodically changing between one of the following four states based on the underlying resource usage algorithms: (i) ‘used’ (e.g, an enzyme is busy in a reaction), (ii) ‘idle’(e.g, an enzyme is free to enter a new reaction), (iii) ‘created’ (e.g, a molecule is created by a reaction) and (iv) ‘decayed’ (e.g, a molecule is in the process of disintegration at the end of its life-cycle). The state transitions are governed by transition flow rates of the dynamic functions involved in it. The process is initiated by a set of input signal(s) from the external world to the system. These input signals initiate the creation of dynamic events which drive the simulation across in time domain, capturing how the system resources change states.

We consider a biological process as a system of resources (typically the various molecules, ions, ribosome-chromosome operon etc involved in the system) that periodically change between one of the following four states (Figure 1) based on the underlying resource usage algorithms: (i) ‘used’ (e.g, an enzyme is busy in a reaction), (ii) ‘idle’(e.g, an enzyme is free to enter a new reaction), (iii) ‘created’ (e.g, a molecule is created by a reaction) and (iv) ‘decayed’ (e.g, a molecule is in the process of disintegration at the end of its life-cycle). The transitions from one state to another are governed by transition flow rates of the dynamic functions involved in the process in a cell. The process is initiated by a set of input signal(s) from the external world to the system. These input signals initiate the creation of dynamic events which drive the simulation across in time domain, capturing how the system resources change states.

**Figure 2:**
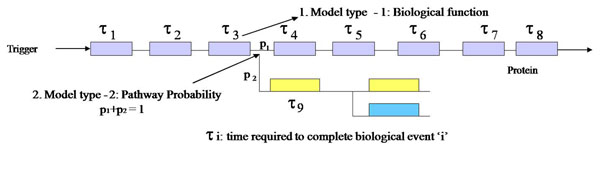
**Modeling scheme for pathway abstraction** Schematic representation of the two types of event models required in our simulation approach: (1) event execution time, and (2) probability of next event type. The figure shows a section of the reaction pathway in a biological process with these two types of events.

Two types of event models are required for this: (1) event execution time, and (2) probability of next event type. Figure 2 shows a section of the reaction pathway of a biological process with these two types of events. A salient feature of this approach is the balance between computational complexity and accuracy of the estimate by including the biological function details as much as possible. The resource interaction between the different events, specially resource conflicts, resource blocking and system dead locks are automatically identified by the simulation. Thus, we can track the important resource counts and identify the exact time of occurrence of these events and direct the next actions based on the outcome. In addition, the simulation can show how the resource dynamics in the system depend on time and external input signal rate, initial values of the resources used in the simulation and the logic of the resource usage algorithms (i.e., the gradual internal changes due to the input signal and the propagation of its effect into the whole system).

**Figure 3:**
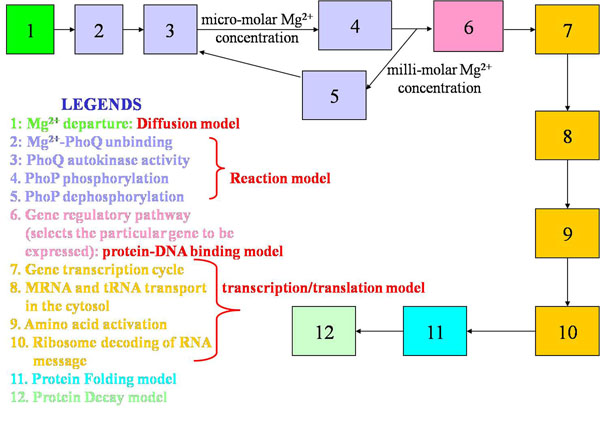
**Biological processes involved in the PhoPQ process in Salmonella** functional event diagram of the PhoP/PhoQ two component regulatory system with the list of discrete events included in the simulation based on the available knowledge of the system.

To illustrate the concept, we present the discrete event modeling of the PhoP/PhoQ two component regulatory system which controls the expression of essential virulence traits in Salmonella Typhimurium depending on the concentration of extra-cellular magnesium [40], [41]. Based on available information, we have developed a functional event diagram (Figure 3) of the process. We identify the list of discrete events that can be included in the simulation based on the available knowledge of the system. In other words, we need to identify the various types of molecules, cells, tissues etc which are involved in the resource usage algorithm for an event (either in reactions, or as catalysts or as end products). To find the time taken for an event, it is important to identify the parameters which affect the interaction of the resources in a particular biological discrete event process and mapping them into the time domain (i.e. identifying the time required for completion of the biological discrete event processing as a function of these parameters). The event holding time algorithms are modeled by stochastic models, diffusion equations and so on.

### PhoPQ biological system model

In Salmonella, virulence is produced by the two-component PhoPQ system that is activated by *Mg*^2+^ concentration change. We identify the key biological functions involved in the PhoPQ regulatory network (from the sensing of *Mg*^2+^ at the cell membrane to the expression of virulent genes in the nucleus). The schematic block diagram of the processes which we have identified to capture the pathway details is presented first. For each process block, we have some input signal(s) coming into the process and output signal(s) which can be considered as the outcome of the process and can trigger one or more processes (or the same process itself in a feedback mechanism). Figure 3 captures the high-level biological functions involved.

#### Mg2+ receptor Signaling Process

Normally a biological process is defined by a pathway (experimentally determined by biologists) that shows the cascade of biological functions in time. Currently, many pathway databases have been established maintaining this record for different species which we use to understand this process. With the departure of a *Mg*^2+^ molecule, the phoQ protein auto-phosphorylates (kinase activity) by making use of an ATP molecule from the cell. The phosphatase activity of phoQ regulates the phosphotransfer mechanism to phosphorylate the phoP protein under micromolar *Mg*^2+^ concentrations, and dephosphorylates the phosphorylated phoP molecules under millimolar *Mg*^2+^ concentrations. Generally, *Mg*^2+^ concentrations higher than 250 mM stimulate the dephosphorylation of phospho-phoP (also called phoPp). Two independent mechanisms of dephosphorylation of phoPp occur. One involves the reversion of the reaction that takes place to phosphorylate the response regulator, and the other is a specific phoPp phosphatase induced by high concentrations of *Mg*^2+^ that renders the release of inorganic phosphate.

Thus we can identify the following discrete events from the PhoPQ pathway: with the departure of a *Mg*^2+^ molecule (event: ion diffusion from membrane protein), the phoQ protein autophosphorylates (kinase activity) by making use of an ATP molecule from the cell (event: membrane reaction). The phosphate activity of the phoQ regulates the phosphotransfer mechanism to phosphorylate the phoP protein under micro molar *Mg*^2+^ concentrations, and dephosphorylates the phosphorylated phoP molecules under millimolar *Mg*^2+^ concentrations (event: cytoplasmic reaction). The Phospho PhoP (phoPp) activates the promoter loci and there is only one activation per phoPp. The loci are obtained from the determination of regulatory pathway. phoPp binding to DNA site is required for transcription (event: DNA protein binding). RNA polymerases are involved in the process of transcription (event: cytoplasmic multi molecule reaction). We also need to consider translation (including steps such as binding of polymerases, regulatory factors, subunits etc) and transport processes.

Thus we can identify many different biological functions and separate models are required to estimate their characteristics. The models for cytoplasmic reactions [42-45], DNA-protein binding [46,47], protein-ligand docking [48,49] and protein synthesis [50] have been reported separately. Here, we present the model for the molecular transport time event. We also explain, how we validate the mathematical model by considering actual molecular data on the PhoPQ system and published experimental results (similar analysis has been done for other model systems e.g., the RNAi pathway in [51]). Based on this model and the other models we mentioned, we can complete the simulation of the PhoPQ system.

### Analytical models for molecular transport

From the PhoPQ system, we find that an important process that we have to model is the movement of molecules (*Mg*^2+^ ions, phoPp etc). We have identified the following movement models for biological processes: (a) diffusion of charged ions (e.g. *Mg*^2+^) in the cell (to model the *Mg*^2+^ arrival/departure process); (b) diffusion of non-charged molecules (to model the transport function of phospho-PhoP in the cytosol); (c) diffusion of charged ions out of the cell (to model the *Mg*^2+^ departure process out of the cell). This movement model should also consider the breakage of the ionic bond between *Mg*^2+^ and phoQ molecules for the diffusion to occur; (d) The fourth movement model is the movement of ions or molecules due to additional energy provided by the pump system. Here, we present the analytical solution of the first two models.

#### Model 1: the diffusion model

**Figure 4:**
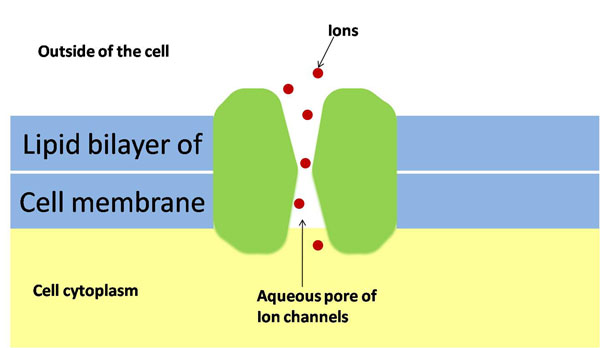
**A simplified illustration of bacterial cell membrane with ion channels** Schematic for the actual diffusion process of *Mg*^2+^ ions inside the cell membrane. The diffusion takes place through an ion-channel at the surface of the cell membrane.

The actual diffusion model of *Mg*^2+^ ions inside the cell membrane is illustrated in Figure 4. The diffusion takes place through an ion-channel at the surface of the cell membrane. In [52], the authors have shown that ion transport through these ion-channels can be appropriately modeled using standard diffusion equations. We consider the following hypothetical mathematical model: suppose that a long capillary (open at one end) filled with water is inserted into a solution of known chemical concentration *C*_0_, and the chemical species diffuses into the capillary through the open end. The concentration of the chemical species should depend only on the distance down the tube and so is governed by the diffusion equation:(1)

where, for convenience, we assume that the capillary is infinitely long. Here, *D* = diffusion constant having units *length*^2^*/time, c* = concentration of the chemical, *t* = time and *x* = distance traversed inside the capillary by the chemical.

Because the solute bath in which the capillary sits is large, it is reasonable to assume that the chemical concentration at the tip is fixed at *C*(0,*t*) = *C*_0_, and because the tube is initially filled with pure water, *C*(*x*,0) = 0.

The solution of this problem is given by [53]:(2)

where . We can compute the *inter-arrival* time between the diffused molecules from the following theorem:

##### Theorem 1

*The inter-arrival time between the diffusion of the* (*i* + 1)*^th^ and i^th^ molecules or ions when the diffusion is based on the concentration gradient only is given by:*(3)

*where I*_*i*+1_*and I_i_ are the times taken for diffusion of the* (*i* + 1)*^th^ and i^th^ molecules respectively, and G is the cross-sectional area of the capillary.*

##### Proof 1

The total number of molecules entering the capillary in a fixed time t is(4)

Thus we get:

It is also possible to determine the diffusion coefficient by solving Eqn 4 for *D*: 

In [54], this expression was used to measure the diffusion constant in bacteria. With concentration *C*_0_ = 7 × 10^7^/ml, and times *t* = 2, 5, 10, 12.5, 15 and 20 minutes, they counted *N* = 1800, 3700, 4800, 5500, 6700 and 8000 bacteria in a capillary of length 32 mm with 1 *μ*l total capacity. In addition, with *C*_0_ = 2.5, 4.6, 5.0, and 12.0 × 10^7^ bacteria per millimeter, counts of 1350, 2300, 3400, and 6200 were found at *t* = 10 minutes. A value of *D* in the range of 0.1 — 0.3 *cm*^2^*/hour* was estimated using Eqn 4.

Also, from Eqn 2 it can be observed that *C*(*x*,*t*)/*C*_0_ is constant on any curve for which *y* is constant. Thus, *t* = *x*^2^/*D* is a level curve for the concentration, and measures how fast the diffusive elements move into the capillary. Here, *t* = *x*^2^/*D* is called the diffusion time for the process. Table 1 shows typical diffusion times for a variety of cellular structures. Clearly, diffusion is quite effective when distances are short, but totally inadequate for longer distances (e.g. along a nerve axon) and biological systems have to employ other transport mechanisms in such situations. For the sample PhoPQ biological system introduced before, the phoPp transport to the cytosol process can be modeled using the diffusion model discussed above. But it is not suited for diffusion of charged molecules, e.g., *Mg*^2+^. Also, this is only an approximate model as the source does not ideally replenish itself. So, we will have better results if the initial concentration *C*_0_ is quite high.

**Table 1:** Estimates of diffusion times for typical cellular structures, computed from the relation *t* = *x*^2^/*D* using *D* = 10^–5^*cm*^2^/*s*

x	t	Example
10 nm	100 ns	thickness of cell membrane
1 *μ*m	1 ms	size of mitochondrion
10 *μ*m	100 ms	radius of small mammalian cell
250 *μ*m	60 s	radius of squid giant axon
1 mm	16.7 min	half-thickness of frog sartorius muscle
2 mm	1.1 h	half-thickness of lens in the eye
5 mm	6.9 h	radius of mature ovarian follicle
2 cm	2.6 d	thickness of ventricular myocardium
1m	31.7 yrs	length of a nerve axon

These diffusion times have been estimated from Model 1.

#### Model 2: diffusion model considering the ion flux

For better analysis of the diffusion process, we need to consider the ion flux through the membrane of width *l* (supposing a potential difference exists across it with *ϕ*(0) = *ϕ*_1_ and *ϕ*(*l*) = *ϕ*_2_) created due to movement of positively charged *Mg*^2+^ ions. We can make a simplifying approximation that the potential gradient through the channel is constant:(5)

If the process is in steady state so that the ion flux everywhere in the channel is the same constant, then the total flux, *J*, can be written as:(6)

where, *α* = *zF/RT*, *z* = total number of positive charges in *Mg*^2+^, *F* = Faraday’s constant, *T* = absolute temperature and *R* = gas constant. Substituting the value of *J* in the diffusion equation we get:(7)

where, *a* = *αV/l*. As it is difficult to achieve a closed form solution of the above equation, we modify the boundary conditions leading to the following theorem:

##### Theorem 2

*The solution to the diffusion problem outlined in Eqn 7 with boundary conditions* 0 <*x* <*l and* > 0 *is given by:*(8)

##### Proof 2

A standard method for obtaining the solution of the above partial differential equation (PDE) is to assume that the variables are separable. Thus we may attempt to find a solution of Eqn 7 by putting

*C* = *Y* (*x*)*Z*(*t*) (9)

where, Y and Z are functions of x and t, respectively. Substitution in Eqn 7 yields(10)

*such that the left hand side depends on t only, and the right hand side depends on x only. Both sides therefore must be equal to the same constant which is conveniently taken as λ*^2^*D. We thus have two ordinary differential equations:*(11)

*Y*″(*x*) + *aY*′(*x*) + *λ*^2^*Y*(*x*) = 0 (12)

The solution for the first equation is:

*Z* = *e*^–^*^λ^*^^2^*Dt*^ (13)

For the second equation, we make a change of variables to bring it down to a standard form as follows:(14)

The solution for f is given by:(15)(16)

where A and B are the constants of integration. Thus we can write:(17)

and the concentration at distance x and time t is given by:(18)

Since we are solving a linear equation, the most general solution is obtained by summing solutions of type Eqn 18 so that we have:(19)

*The previous capillary model cannot be used in this case to obtain a solution because the underlying complexity becomes immense. We will now consider diffusion out of a plane sheet of thickness l through which the diffusing substance is initially uniformly distributed and the surfaces of which are kept at zero concentration. Mapping this model to our case, the ion channel of length l is assumed to contain the entire diffusing substance. Every single molecule coming out of this sheet is assumed to enter the cell membrane (Mg*^2+^*arrival process). This model thus approximately characterizes the Mg*^2+^*diffusion process. The corresponding boundary conditions are as follows:*

*C*(*x*,0) = *C*_0_, 0 <*x* <*l* (20)

*C*(0,*t*) = 0, *C*(*l*,*t*) = 0 (21)

where Eqn 20 signifies the initial concentration inside the ion channel and Eqn 21 signifies the initial concentration (before the start of diffusion) inside the cell membrane. Eqn 21 yields:

*Also, substituting B_m_* = 0 *in Eqn 21 for x* = *l, we get:*(22)

The solution can be obtained by elimination of variables such that we have:

Substituting these values in Eqn 20 we get:(23)

*Multiplying both sides of Eqn 23 by**and integrating with respect to* 0 *to l, we get:*(24)

We will make use of the following identities for the solution of A_m_:(25)

Substituting these identities in Eqn 24, we get:(26)

And hence we can write:(27)

Thus we get the time domain analysis for the concentration of *Mg*^2+^ molecules from which we can derive the mean *Mg*^2+^ departure rate. The inter-arrival time between the diffused molecules can be computed from the following theorem:

##### Theorem 3

*The inter-arrival time between the diffusion of the* (*i* + 1)*^th^ and i^th^ molecules or ions when the diffusion is based on both the concentration and potential gradients across the cell is given by I_N–i_ – I_N–i–1_, where I_N–i_ and I_N–i–1_ are the times taken for diffusion of the i^th^ and* (*i* + 1)*^th^ molecules/ions respectively and can be solved from the following equations:*(28)(29)

##### Proof 3

*The total number of molecules/ions, N, present inside the sheet of area G in a fixed time I_N_ is given by:*

The inter-arrival time can be computed in a straight-forward way by noting that diffusion occurs when a molecule/ion goes out off the plane sheet.

The third diffusion model basically characterizes reaction-diffusion systems and can be simply computed by convoluting the event time distributions (which are random variables) of a reaction model and any of the above diffusion models.

### Numerical results for the molecular transport models

Here we present the numerical results for our diffusion models and Table 2 concisely presents the parameters used.

**Table 2:** Parameter Estimation for the numerical plots

Parameters	Salmonella cell
Diameter of an ion-channel (d)	10 × 10^–10^*m*
Cross-sectional area of ion-channel (*G*′)	
Number of ion-channels (*N*′)	100
*G*	*N*′ × *G*′
*D*	10^–5^*cm*^2^/*s*
*V*	60 *mV*

List of parameters used to generate the numerical results on the two diffusion models reported in the paper.

**Figure 5:**
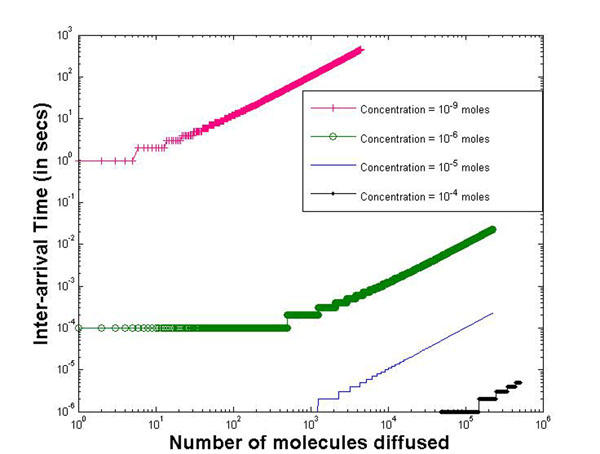
**Inter-arrival time vs number of molecules for diffusion model 1** Inter-arrival time of diffused molecules for molecular concentrations ≃ 10^–9^, 10^–6^, 10^–5^, 10^–4^ moles respectively governed by Model 1. This model as stated earlier is suitable for diffusion of uncharged molecules. The figure shows that the inter-arrival time increases with increasing number of molecules diffused in. This is because the concentration gradient reduces with more molecules diffusing in, resulting in larger time required for the molecules to move in. Also, it is observed that larger the initial concentration, the lesser is the inter-arrival time. This is expected due to a higher concentration gradient. This inter-arrival time distribution can be easily fitted to an exponential distribution.

Figure 5 plots the inter-arrival time of diffused molecules for molecular concentrations ≃ 10^–9^,10^–6^,10^–5^,10^–4^ moles respectively governed by Model 1. This model as stated earlier is suitable for diffusion of uncharged molecules. The figure shows that the inter-arrival time increases with increasing number of molecules diffused in. This is because the concentration gradient reduces with more molecules diffusing in, resulting in larger time required for the molecules to move in. It is observed that larger the initial concentration, the lesser is the inter-arrival time. This is expected due to a higher concentration gradient. Also, it can be observed that the inter-arrival time distribution can be fitted to an exponential distribution.

**Figure 6:**
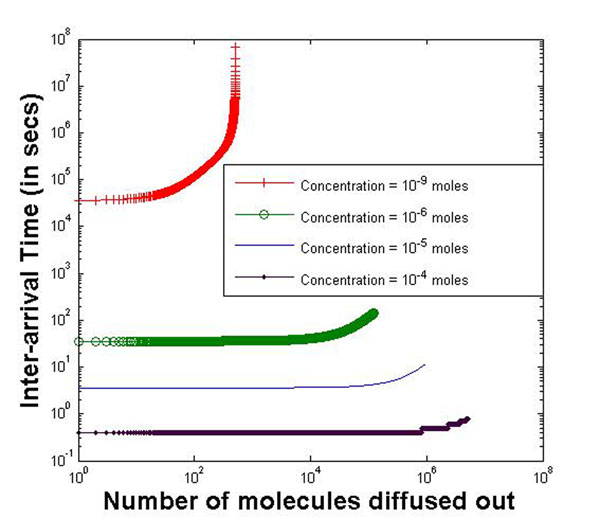
**Inter-arrival time against number of molecules for diffusion model 2** Inter-arrival times for diffusion model 2 where the potential gradient is considered. We assume a constant potential gradient of 60mV for the molecules to overcome for diffusion to take place. The inter-arrival times are higher than the first model because the molecules have to overcome the potential gradient as well in order to diffuse. Here, the exponential increase in the inter-arrival times can be observed more clearly. This scenario is best depicted by the curve for concentration 10^–9^*moles* where the results are generated for a large number of molecules diffusing out.

Figure 6 plots the inter-arrival times for diffusion model 2 where the potential gradient is considered. We assume a constant potential gradient of 60mV for the molecules to overcome for diffusion to take place. The inter-arrival times are higher than the first model because the molecules have to overcome the potential gradient as well in order to diffuse. Here, the exponential increase in the inter-arrival times can be observed more clearly. This scenario is best depicted by the curve for concentration 10^–9^*mole*s where the results are generated for a large number of molecules diffused out.

Note that model 1 is standard and we estimated the inter-arrival times of *Mg*^2+^ molecules using it. The transient analysis of model 2 is hard to solve and hence we chose a specific boundary condition (as mentioned before) to derive a closed form expression. The corresponding results compare well with model 1 indicating its validity.

### Simulation results of the PhoPQ system

As the arrival/departure of *Mg*^2+^ molecules into the cell membrane is essentially a stochastic process, a constant diffusion rate is not suitable to trigger the input process of the PhoPQ system. Hence we use an *exponential distribution* (as indicated by the numerical plots above) to estimate the inter-arrival times for diffusion of *Mg*^2+^ (which is considered to be a random variable) to generate the results. The mean of this exponential distribution is obtained from similar plots of inter-arrival times as shown above and corresponding curve-fitting. As mentioned before, the PhoPQ system is triggered at micromolar concentrations of *Mg*^2+^ outside the cell, i.e., with millimolar *Mg*^2+^ concentration inside the cell. Thus it is fair to assume *C*_0_ ≃ 10^–3^ moles. The mean of the inter-arrival times of *Mg*^2+^ for this concentration is estimated to be ≈ 10^–6^ secs for Model 1 and 10 msecs for Model 2 respectively. The discrete-event simulation framework correspondingly uses a Poisson distribution with the same mean (as the inter-arrival times follow an exponential distribution) to estimate the time taken for the departure process of *Mg*^2+^triggering the signal transduction cascade (following Model 2) and an exponential distribution to estimate the phoPp molecule transport times (following Model 1).

The simulation framework also uses the holding time estimates of other elementary biological processes such as cytoplasmic reactions [42], [43], [44], [45] (models 2, 3, 4 and 5 in Figure 3), protein-DNA binding [46] (model 6 in Figure 3) and gene transcription/translation times [50]. Here, we present the results illustrating the sensitivity of the simulation to the diffusion models used.

#### Modeling validation and performance measurement

The efficacy of an in silico modeling and simulation approach is governed by

(a) validation of the model against existing wet-lab experimental results,

(b) effective calibration and sensitivity analysis of the key parameters governing the biological model and

(c) hypothesis testing of different conditions on the biological system which can give further insights for novel experiments in the future.

In this section, we employ the discrete event based stochastic simulation framework to model the dynamics of single cell dynamics, specifically, the effect of the PhoPQ two-component signal transduction pathway on the expression of virulence genes involved in bacterial pathogenesis of the gram-negative bacteria Salmonella Typhimurium. While the simulation system can be used to model the temporal dynamics of different regulatory pathways in a bacterial cell, we focus on the particular system in this work as it provides,

1. Existing wet-lab experimental setup and results [55] which allow the validation of the in silico results

2. The system involves the interaction of signal transduction with subsequent expression of genes governed by the upstream signals

3. The gene regulation pathway as built based on existing literature on the two-component system provides various regulatory mechanisms including up and down regulation of genes, and positive feedback effects which can serve to test different hypothesis in silico.

4. As the system involves complex biological functions like gene regulation and protein expression, whose exact molecular mechanisms are not always well known, it provides a platform to test the efficacy of granular model abstraction based on available knowledge, on the behavior at a systems level.

In the rest of the section, we start with a brief description of the wet lab experimental system, moving on to present the detailed results of in silico analysis. We show how the discrete event simulation framework can be used for hypothesis-driven analysis of different conditions in silico for the PhoPQ system.

#### In-silico model validation with wet lab experimental system

The experimental setup, explained in details in [55], consists of reporting the system output of the phoPQ pathway on bacterial cells. As reported in [55], fluorescence measure of expression of destabilized green fluorescence protein (dEGFP) under the control of a phoPp (phosphorylated phoP) responsive promoter was used as the reporter system. Thus, the system measure of the dEGFP was in essence an indication of the phoPp concentration in the system.

**Figure 7:**
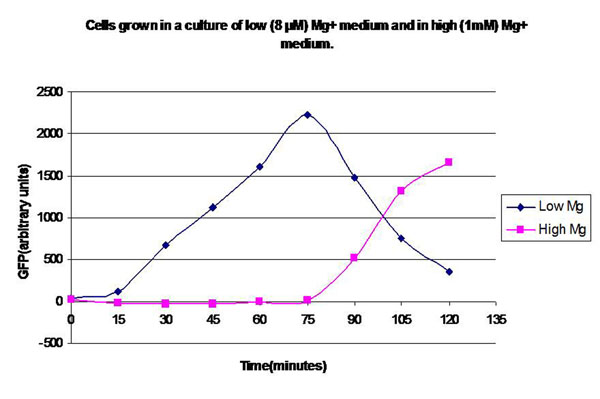
**Effect of *Mg*^2+^ on the system output (measured by the surrogate marker dEGFP** Experimental plot showing the system output of the cell culture in time, both for high-magnesium as well as low-magnesium conditions.

**Figure 8:**
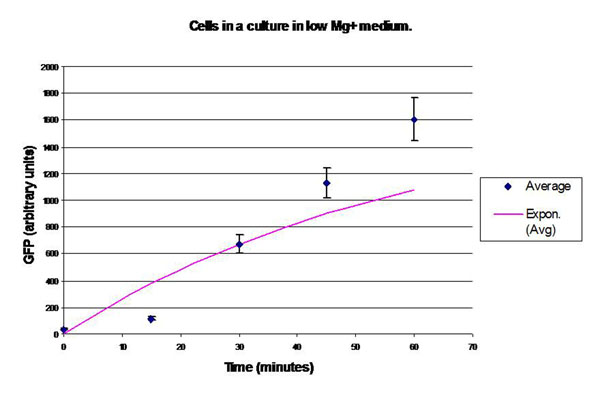
**Effect of low *Mg*^2+^ (8*μ*M) on the system output (measured by the surrogate marker dEGFP** Experimental plot showing the system behavior as observed for time of 60 mins when the cells were in a culture of low (8*μM*) magnesium medium. It shows how in low magnesium, the PhoPQ pathway is activated (as shown by increase in concentration of phoPp).

**Figure 9:**
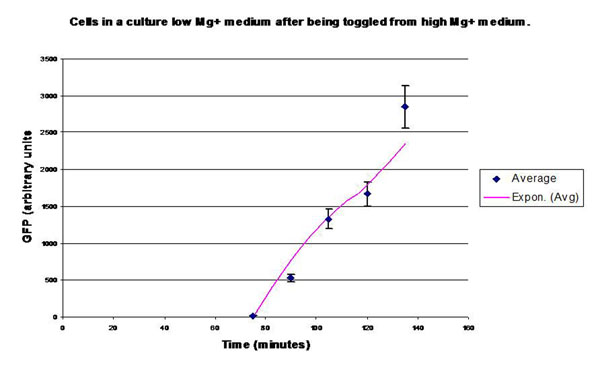
**Effect on the system output when toggled from high to low *Mg*^2+^ concentration** Experimental plot showing the toggling effect of the ‘on-off’ switch mechanism when the system state was changed from high to low magnesium medium.

In the experimental system, low *Mg*^2+^ was maintained for a period of 60 mins, during which the system output increased, after which the signal was toggled to high *Mg*^2+^. The measurements of the fluorometer were taken every 15 mins for the positive activation state. Figure 7 shows the system output of the cell culture in time, both for high-magnesium as well as low-magnesium conditions. Figure 8 shows the system behavior as observed for time of 60 mins when the cells were in a culture of low (8*μM*) magnesium medium. It shows how in low magnesium, the PhoPQ pathway is activated (as shown by increase in concentration of phoPp). Similarly, Figure 9 shows the toggling effect of the ‘on-off’ switch mechanism when the system state was changed from high to low magnesium medium. Based on these experiments, we run the discrete event simulation to generate in silico results which capture the system output in time. The simulation initialization with different resource and system parameters are key to the success of the model.

Also, the platform provides flexibility in changing these conditions and resources to generate synthetic, hypothetical results for a better understanding of the test system. In the next subsection, we outline the system and simulation parameters and present the results of the in silico experiment.

**Figure 10:**
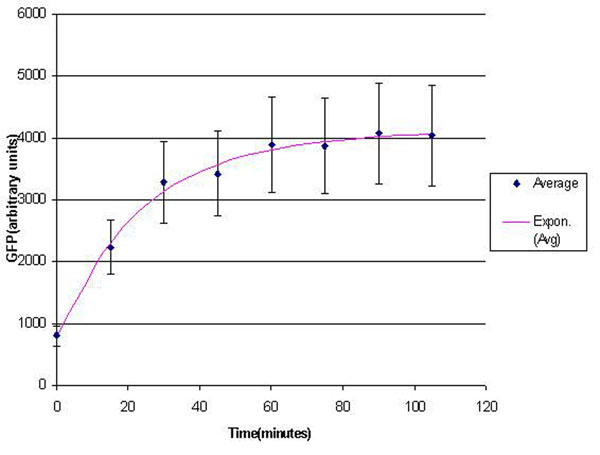
**Experimental results: concentration of phoPp molecules with time with *Mg*^2+^concentration 10^–3^ moles** Experimental plot for the concentration of phoPp molecules against time as observed in wet lab experiments [55].

Figure 10 plots the concentration of phoPp molecules against time as observed in wet lab experiments [55]. At present it is difficult to directly link the results of the simulation to the wet lab experiments data that we have. This is because simulation gives the temporal dynamics in actual molecular count, whereas the fluorescent tag based wet lab experiments only show the sensitivity of the fluorescent light. It was not possible to calibrate the fluorescent tag sensitivity to molecular count per cell in the past. Thus our simulation results validate the similarity of the temporal dynamics of experimental results now, without actual comparison of the molecular count of a cell. Currently more sophisticated experiments like microfluidic based single cell assay [56] allows real time observation of single molecules in a cell. In future, we hope to get molecular level measurements in a cell to validate our results quantitatively.

##### Simulation setup

Next, we setup the ‘dry-lab’ experimental system for the signal transduction and subsequent gene regulation pathway involved in the test-bed. The in silico experiment is initialized with the system molecular resources and biological parameters associated with the probability distribution functions of the different event holding time modules. In this experiment, we focused on parameters associated with the Salmonella bacterial cell based on the CCDB database [27] which are summarized in Table 3. The simulation also initializes other resource parameters like the number of molecules (in terms of concentration) for the different species involved in the system (e.g. ATP, ADP, phoP, phoQ, extracellular *Mg*^2+^ ions) and the gene regulatory pathway information extracted during the PhoPQ pathway creation phase. Once the system is initialized, the event queue is populated with the initial event list which determines the snapshot of the biological environment at simulation start time and the simulation engine is triggered.

**Table 3:** System model and simulation parameters

Biological Parameters	Value
Length of Genome	4857432
Number of Genes	4451
Rate of transcription	40 nucleotides/sec
Rate of translation	18 residues/sec
Area of cell	6 × 10^–12^*m*^2^
Volume of cell	10^–18^*m*^3^
Diffusion coefficient of *Mg*^2+^ ion	10^–9^*m*^2^/*s*
Diffusion coefficient of protein molecule	7.7 × 10^–6^*m*^2^/*s*
Average mass of a protein molecule	25 kDa
Average diameter of a protein molecule	5 nm

List of parameters used to generate the complete discrete event based simulation results

**Figure 11:**
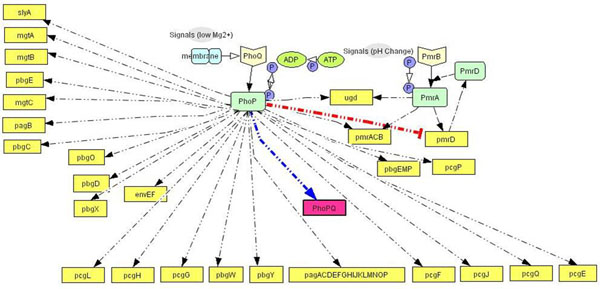
**PhoPQ gene regulatory pathway** Manually-curated comprehensive gene regulatory pathway network for the phoPQ system. The two component pathway involves transcriptional regulation of 44 genes, 5 of which are involved in another cascading two component system (pmrBA). A positive feedback loop exists in this pathway, in the form of up regulation of phoPQ gene by the system. The figure shows the complete pathway, with the positive feedback loop marked in dark color. The pathways have been constructed using the Cell Designer 3.0 software which presents a structured (Extensible Markup Language (XML)) format data that can be easily rendered into the discrete event simulation framework. The process involved 112 experimental reports of this system extracted from PubMed [57], development of the pathway graphs for each experiment and then concatenating those graphs to get the complete pathway graph.

We used comprehensive knowledge extraction from PubMed database [57], to construct the gene regulatory pathways for the phoPQ network, identifying the common intersection of the pathways i.e. the genes and gene products which are regulated by this system at various stages. In our current work, the two component pathway involves transcriptional regulation of 44 genes, 5 of which are involved in another cascading two component system (pmrBA). A positive feedback loop exists in this pathway, in the form of up regulation of phoPQ gene by the system. Figure 11 shows the complete pathway, with the positive feedback loop marked in dark color. The pathways have been constructed using the Cell Designer 3.0 software which presents a structured (Extensible Markup Language (XML)) format data which can be easily rendered into the discrete event simulation framework. The process involved 112 experimental reports of this system extracted from PubMed [57], development of the pathway graphs for each experiment and then concatenating those graphs to get the complete pathway graph.

For the current system, the simulation focused on tracing the effects of signaling events (*Mg*^2+^ ion arrival and departures) on the expression dynamics of the PhoPQ pathway. Also, as a reporter protein (GFP) has been used in the wet-lab scenario to trace the system behavior, our results are focused primarily on phoPp as the main resource whose dynamic temporal behavior was observed in the simulation. Although, the simulation can be configured to monitor and generate results for a wide range of system resources, phoPp was chosen primarily to verify the wet-lab tests. The in-silico results denote resource states averaged over 100 runs of the simulation under the same initial conditions.

**Figure 12:**
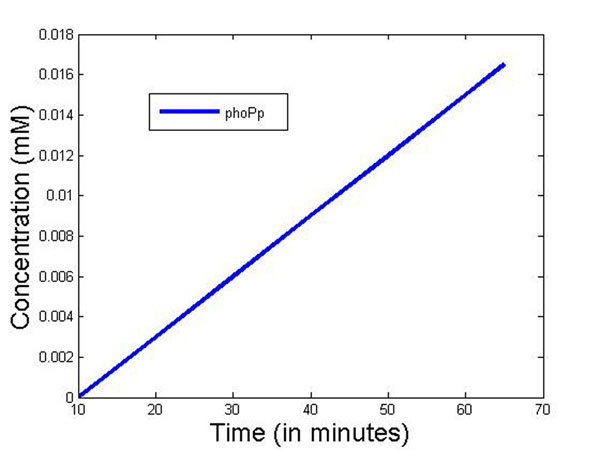
**Effect of low *Mg*^2+^ on the in silico system** The simulation was configured to run with low *Mg*^2+^ for 60 mins, during which no resource conflicts or starvation were assumed (i.e, the simulation would not stop due to lack of any resource). As seen in this figure, the simulation responds with continuous growth in phoPp concentration, implying increasing dEGFP fluorescence.

In order to simulate similar conditions “in silico”, the simulation was configured to run with low *Mg*^2+^ for 60 mins, during which no resource conflicts or starvation were assumed (i.e, the simulation would not stop due to lack of any resource). As seen in Figure 12, the simulation responds with continuous growth in phoPp concentration, implying increasing dEGFP fluorescence.

**Figure 13:**
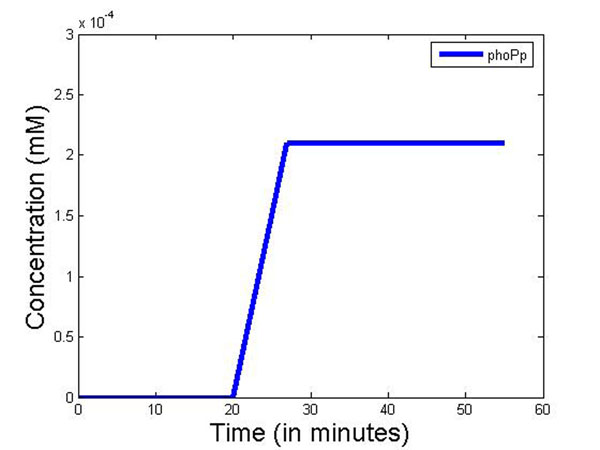
**In silico system output when *Mg*^2+^ conc. changes from high to low** The simulation was started with high *Mg*^2+^ which was switched to low *Mg*^2+^ at 20 mins and was kept low for 30 mins. and toggled back to high after that. This figure captures the system response under this scenario.

In another simulation experiment, the system was started with high *Mg*^2+^ which was switched to low *Mg*^2+^ at 20 mins which was kept low for 30 mins. and toggled back to high. Figure 13 captures the system response under this scenario. As seen from these figures, the effects captured by the simulation show similar dynamics to the wet-lab system.

**Figure 14:**
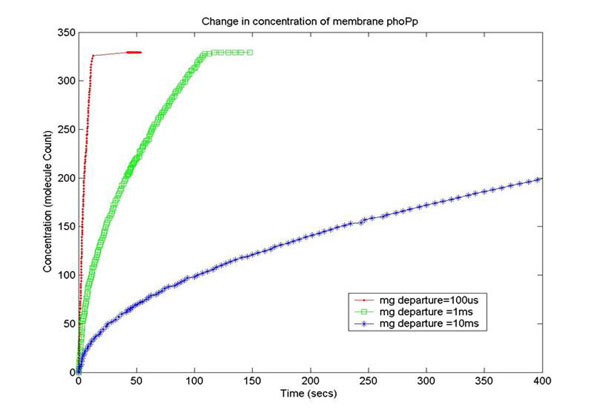
**Simulation results: concentration of phoPp molecules with time with *Mg*^2+^ concentration 10^–3^ moles** Simulation plots for the phoPp concentration change with 3 different means for the *Mg*^2+^ departure process. It can be noted that with mean = 100*μs*, the phoPp concentration change is quite steep, and it achieves the maximum value of phoPp (observed experimentally) in the cell at ≈ 1 sec. But as the mean is increased to 10 ms, we get acceptable estimates of the phoPp concentration. This outlines the importance of diffusion Model 2 where the mean of the *Mg*^2+^ departure process is indeed in the range of 10 ms as against the 1*μ*s range for Model 1.

Figure 14 plots the phoPp concentration change from our discrete event simulation framework with 3 different means for the *Mg*^2+^ departure process. It can be noted that with mean = 100*μ*s, the phoPp concentration change is quite steep, and it achieves the maximum value of phoPp (observed experimentally) in the cell at ≈ 1 sec. But as the mean is increased to 10 ms, we get acceptable estimates of the phoPp concentration. This outlines the importance of diffusion Model 2 where the mean of the *Mg*^2+^ departure process is indeed in the range of 10 ms as against the 1*μ*s range for Model 1. As discussed earlier, Model 1 is suitable for the phoPp transport process in the cytosol.

Also, the condition of no resource starvation shows relative smoothness in output as obtained from continuous system models since the effect of low copy number of molecules on stochasticity [16] is not displayed. The in silico platform allows the analysis of the effects of stochasticity on the model by varying the resource states of the molecules involved in the simulation and also the sensitivity of the system outputs to the different parameters governing the event holding time distributions. In the next sub-section, we present a systematic analysis of the different in silico hypothesis tests.

#### In silico hypothesis testing

The in-silico simulation allows the modeler to test the system under various synthetic conditions, in terms of system resource states, initial conditions and different combinations of environmental cues driving the systems (for example, the diffusion of *Mg*^2+^ through the cell membrane in our case study).

**Figure 15:**
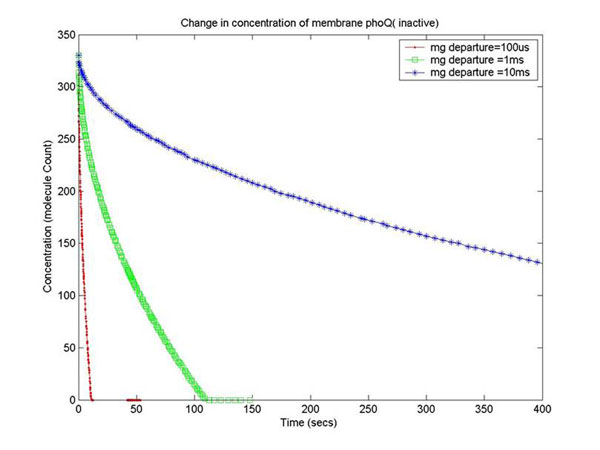
**Change in conc. of membrane phoQ** In order to capture the effects of varying the rate of diffusion of *Mg*^2+^ on the system output, the simulation was ran with increasing *Mg*^2+^ diffusion rates (with means of 100ms, 1ms and 10ms). The results have been reported for two key system resources, the proteins phoQ, which is the sensory protein responsible for binding to *Mg*^2+^, and the phoP protein, which controls the dynamics of gene expression. The figure shows how the rate of decrease in the concentration of inactive phoQ (phoQ molecule bound to a *Mg*^2+^) is damped with increasing delay in the diffusion of *Mg*^2+^ out of the membrane. Also, captured in this graph is the effect of resource starvation on the biological system. As the *Mg*^2+^ ion initiated signal activates the PhoPQ pathway, the sensory phoQ proteins are consumed by the system, thereby shutting down the pathway when all phoQ molecules available to the system have been used.

**Figure 16:**
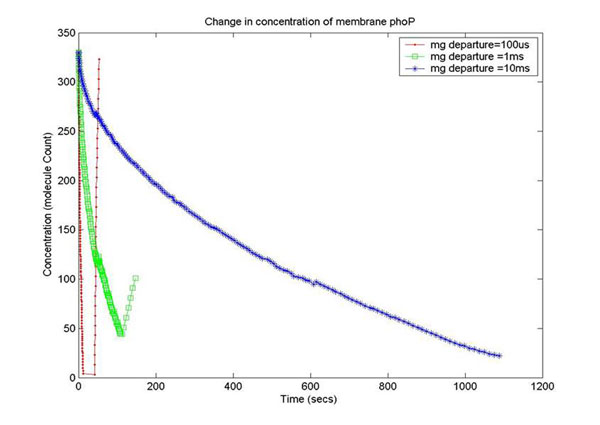
**Change in conc. of membrane phoP** Simulation plot capturing the effects of varying diffusion rates on phoP. An interesting observation, not captured in the wet-test lab results, is the orchestration of the positive feedback loop of phoP, as identified in the knowledge extraction phase. As seen in this figure, the concentration of phoP in the system decreases initially; but once the expression of genes is triggered by phoPp (phosphorylated phoP), phoP starts appearing in the system. The corresponding effect on phoPp, which increases in concentration when *Mg*^2+^ depart from the membrane (activating the pathway) is captured in Figure 14. In both the graphs, the slowest diffusion rate does not bring the system into resource shortage phases while the other diffusion rates lock the system (plateau on Figure 14) due to non-availability of phoPp molecules. These graphs show how the tuning of different parameters (in the diffusion rates) can be synthetically manipulated to study different behaviors of the systems.

In order to capture the effects of varying the rate of diffusion of *Mg*^2+^ on the system output, we ran the simulation with increasing *Mg*^2+^ diffusion rates (with means 100ms, 1ms,10ms) and reported the results for two key system resources, the proteins phoQ, which is the sensory protein responsible for binding to *Mg*^2+^, and the phoP protein, which controls the dynamics of gene expression. Figure 15 shows how the rate of decrease in the concentration of inactive phoQ (phoQ molecule bound to a *Mg*^2+^*)* is damped with increasing delay in the diffusion of *Mg*^2+^ out of the membrane. Also, captured in this graph is the effect of resource starvation on the biological system. As the *Mg*^2+^ ion initiated signal activates the PhoPQ pathway, the sensory phoQ proteins are consumed by the system, thereby shutting down the pathway when all phoQ molecules available to the system have been used. Similarly, Figure 16 captures the effect of the same conditions on phoP. An interesting observation, not captured in the wet-test lab results, is the orchestration of the positive feedback loop of phoP, as identified in the knowledge extraction phase. As seen in Figure 16, the concentration of phoP in the system decreases initially; but once the expression of genes is triggered by phoPp (phosphorylated phoP), phoP starts appearing in the system. The corresponding effect on phoPp, which increases in concentration when *Mg*^2+^ depart from the membrane (activating the pathway) is captured in Figure 14. In both the graphs, the slowest diffusion rate does not bring the system into resource shortage phases while the other diffusion rates lock the system (plateau on Figure 14) due to non-availability of phoPp molecules. These graphs show how the tuning of different parameters (in the diffusion rates) can be synthetically manipulated to study different behaviors of the systems.

The in silico results on the test-bed pathway demonstrate the efficacy of the modeling and simulation approach for studying single cell dynamics. Particularly, the flexibility in event scheduling and resource state specifications allows a modeler to validate the effects of high and low copy number of molecules on different parts of the biological system. Moreover, the flexibility allows the simulation to be computationally efficient depending on the required granularity of the biological model and the resource state space considered [58].

## Conclusion

We have proposed a new “in silico” modeling technique capturing the temporal dynamics of biological systems at multiple scales that can be simulated by the discrete event technique. For this, we need the transformation of biological functions into information theory based measure like probability distributions of event time. We have presented one example of the transformation of a biological function (i.e., molecular transport time) driven by concentration and potential gradients in this paper. We also validated the molecular transport models and put together a discrete event simulation for the PhoPQ system to validate the system level dynamics based results with experimental estimates. We also used this molecular transport model to generate some in-silico hypothesis testing results on the PhoPQ system.

The proposed stochastic models meet the accuracy and computational speed requirements for modeling complex biological processes. These models are parametric and can be used for different cases of molecular transport. Once the complete set of mathematical models for the different biological functions are in place, it should be possible to reuse these models to construct other biological process models with marginal changes. The models provide for both speed of computation and flexibility that is required to model the dynamics of an entire cell. We envisage the development of an efficient tool for understanding the dynamics of complex biological systems that can model the multi-scale biological process at a coarse grain accuracy.

## Competing interests

The authors declare that they have no competing interests.

## Authors contributions

PG helped with the development of the model and simulation and generating the results. PG, SG, KB, SKD and CZ helped with conceptualizing the whole project and writing the paper. All authors have read and approved the paper.
